# Versatile *in situ* powder X-ray diffraction cells for solid–gas investigations

**DOI:** 10.1107/S0021889810038148

**Published:** 2010-10-20

**Authors:** Torben R. Jensen, Thomas K. Nielsen, Yaroslav Filinchuk, Jens-Erik Jørgensen, Yngve Cerenius, Evan MacA. Gray, Colin J. Webb

**Affiliations:** a Center for Energy Materials, Center for Materials Crystallography, iNANO and Chemistry Department, University of Aarhus, Langelandsgade 140, DK-8000 Aarhus C, Denmark; b Swiss–Norwegian Beam Lines, European Synchrotron Radiation Facility, 38043 Grenoble, France; c MAX-lab, Lund University, S-22100 Lund, Sweden; d Queensland Micro- and Nanotechnology Centre, Griffith University, Nathan 4111, Brisbane, Australia

**Keywords:** powder X-ray diffraction, X-ray diffraction cells, solid–gas reactions, hydrogen absorption

## Abstract

This paper describes new sample cells and techniques for *in situ* powder X-ray diffraction specifically designed for gas absorption studies up to *ca* 300 bar (1 bar = 100 000 Pa) gas pressure. The cells are for multipurpose use, in particular the study of solid–gas reactions in dosing or flow mode, but can also handle samples involved in solid–liquid–gas studies. The sample can be loaded into a single-crystal sapphire (Al_2_O_3_) capillary, or a quartz (SiO_2_) capillary closed at one end. The advantages of a sapphire single-crystal cell with regard to rapid pressure cycling are discussed, and burst pressures are calculated and measured to be ∼300 bar. An alternative and simpler cell based on a thin-walled silicate or quartz glass capillary, connected to a gas source *via* a VCR fitting, enables studies up to ∼100 bar. Advantages of the two cell types are compared and their applications are illustrated by case studies.

## Abstract 2

Two multipurpose sample cells of quartz (SiO_2_) or sapphire (Al_2_O_3_) capillaries, developed for the study of solid–gas reactions in dosing or flow mode, are presented. They allow fast change of pressure up to 100 or 300 bar (1 bar = 100 000 Pa) and can also handle solid–liquid–gas studies.

## 1. Introduction

Understanding the processes by which different materials absorb gas, *e.g.* hydrogen, is critical to the further development of materials and additives to improve thermodynamics, kinetics, gas separation and storage capacity. For metallic hydrides it is important to understand the different phases and their behaviour as a function of hydrogen content, as well as the role of vacancies and dislocations (*e.g.* Kisi *et al.*, 1994 ▶; Wu *et al.*, 2002 ▶). For complex hydrides and nanoconfined hydrides it is important to understand the intermediate reaction products, their stability and the conditions under which they form, as well as the role of additives and their function and distribution (Nielsen *et al.*, 2010 ▶; Bösenberg *et al.*, 2010 ▶). In order to gather data as a function of hydrogen absorption it is necessary to perform *in situ* experiments. X-ray diffraction is an informative analysis technique for crystalline materials, which include most of the solid hydrides. In addition, X-ray scattering typically requires a shorter data-acquisition time than neutron diffraction and is therefore more amenable to kinetic studies of hydrogen absorption and desorption. Combined with the ability of the experimental arrangement described here to change the hydrogen pressure over more than five orders of magnitude within *ca* 10 s, excellent time resolution of hydrogen absorption and desorption reactions can be obtained.

Hydrogen absorption and desorption reactions are typically associated with considerable change in unit-cell volume, often of >20% for interstitial metallic hydrides, whereas ionic hydrides often change from ionic to metallic form, and covalently bonded complex hydrides also significantly change structure and composition (Andreasen *et al.*, 2005 ▶; Nielsen *et al.*, 2009 ▶; Kisi *et al.*, 1994 ▶). Solid crystalline materials are usually involved as both reactants and products in these reactions. Therefore, even though hydrogen has the lowest X-ray scattering power of any element, the effects of hydrogen absorption and desorption are easily observable (Hauback, 2008 ▶; Filinchuk, Chernyshov & Dmitriev, 2008 ▶; Gray *et al.*, 2006 ▶; Orimo *et al.*, 2007 ▶; Züttel, 2004 ▶).

Here, we present two sample cells for *in situ* diffraction studies under variable gas pressure. One, (A), is routinely used in the research laboratory MAX-lab at the MAX II synchrotron, Lund, Sweden, and is based on a single-crystal sapphire capillary, which allows loading 300 bar of gas (1 bar = 100 000 Pa). The other, (B), is used at the Swiss–Norwegian Beamlines (SNBL) at the European Synchrotron Radiation Facility (ESRF), Grenoble, France, and employs thin-walled glass or quartz capillaries and is used at pressures up to 100 bar. Both sample cells allow the collection of high-quality powder and single-crystal diffraction data.

## 2. Equipment design and implementation

### 2.1. Setup for *in situ* experiments

The experimental setup for the sapphire-based cell (A) is implemented at the MAX II synchrotron in the Swedish national research laboratory MAX-lab at beamline I711 (Cerenius *et al.*, 2000 ▶). The selected X-ray wavelength is typically in the range 1.0–1.1 Å. Beamline I711 is equipped with a Marresearch MarCCD 165 area detector, which has been successfully used for X-ray powder diffraction experiments. The cell employing thin-walled capillaries, (B), is routinely used at the Swiss–Norwegian Beam Lines (SNBL) at the ESRF. A Marresearch MAR345 image-plate detector and wavelengths from 0.65 to 0.85 Å are typically used there.

The use of an area detector gives several advantages. Firstly, integration of the intensity from the complete X-ray diffraction cone can reduce or eliminate preferred orientation effects. Secondly, when chemical reactions occur in the reaction cell, the particle size of the product may be significantly higher than that of the reactants, giving more single-crystal-like (‘spotty’) data, instead of the smooth diffraction rings produced by the fine-powder reactants. Thus, area detectors contribute to the improvement of the powder average. Phase analysis is also simplified when the phases can be distinguished on the basis of their azimuthal distribution. Thirdly, diffraction from sources other than the sample can often be visually detected, either as diffraction rings with a centre different from the direct X-ray beam or as diffraction spots located away from the diffraction rings from the sample. The program *FIT2D* (Hammersley, 1997 ▶) is used to convert the area-detector frames (image files, binary format) to powder diffraction patterns, *I*(2θ) (ASCII format), and to mask out unwanted diffraction, *e.g.* from a sapphire sample holder (Hammersley *et al.*, 1996 ▶). Sapphire cells may produce up to a few sharp diffraction spots originating from the single-crystal sapphire capillary. These diffraction spots may overexpose the detector but they are conveniently removed by rotating the entire sample cell, *e.g.* by Δϕ = ±2°, in order to avoid the diffraction condition from the sapphire crystal. Diffraction spots from sapphire must also be masked during data integration.

### 2.2. Design of the sapphire-based cell

**Figure 1:**
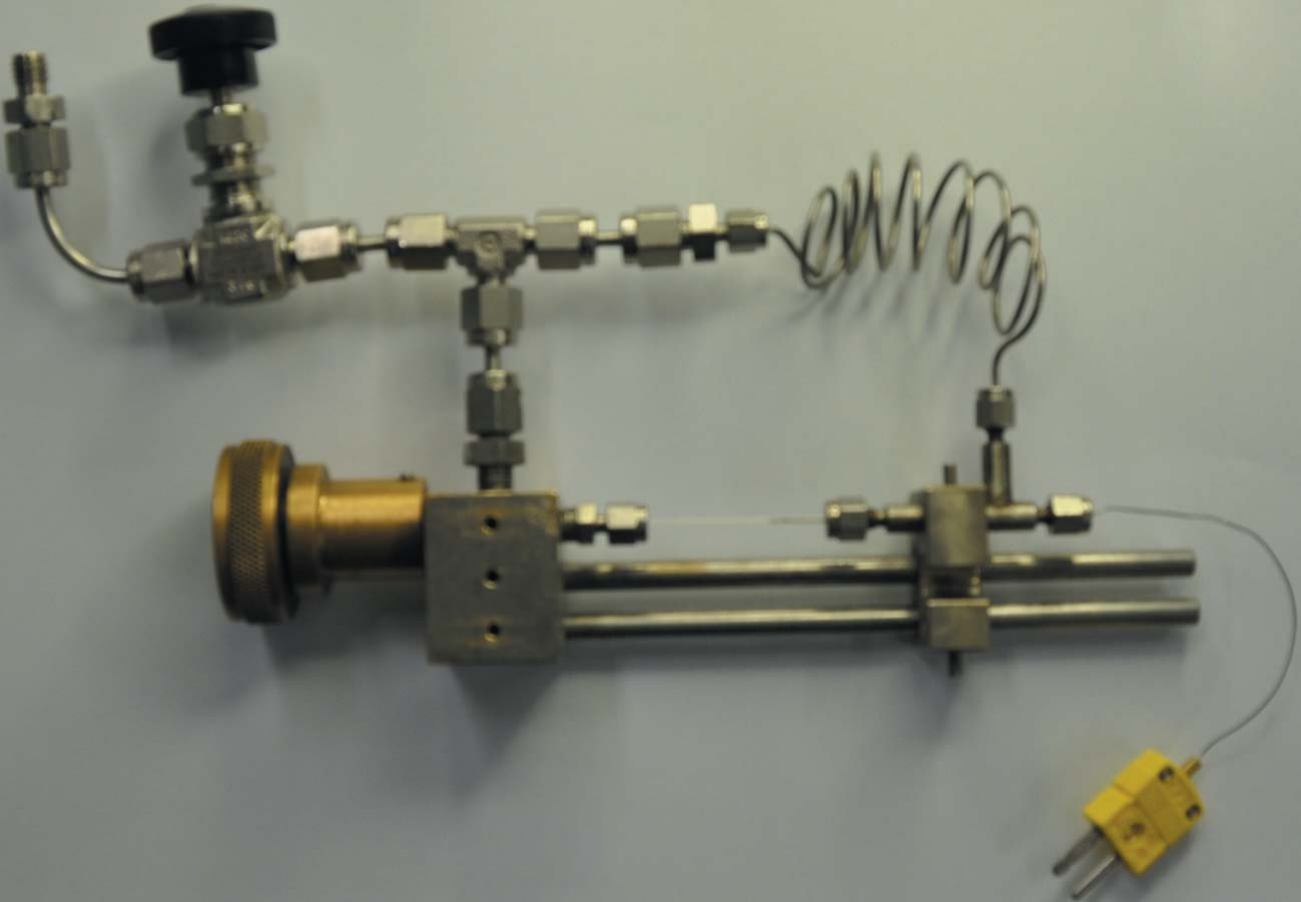
The cell based on a sapphire capillary, (A). A flexible stainless steel capillary connects the two ends of the sample holder and allows dosing of the sample with a selected gas from both ends simultaneously, and also allows for an easy and fast change of sample.

The sample cell (A), shown in Fig. 1 ▶, is a modification of previously described sample cells designed mainly for studies of catalytic reactions utilizing a flow of gas through a powdered sample (Clausen *et al.*, 1991 ▶; Chupas *et al.*, 2001 ▶, 2008 ▶; Tonus *et al.*, 2009 ▶). The sample is held in a single-crystal sapphire (Al_2_O_3_) capillary, 1.09 mm outer diameter and 0.79 mm inner diameter, supplied by Saint-Gobain Crystals, USA (http://www.photonic.saint-gobain.com/). The capillaries are grown so that the longitudinal direction coincides with the crystallographic *c* axis. Sapphire is one of the hardest materials in existence (9 on the Mohs scale), is virtually scratch proof and has a maximum working temperature of *ca* 2273 K (it melts at 2326 K), making it ideal for high-temperature applications. According to the manufacturer, it is chemically inert to hydrofluoric acid and fluorine plasma. Sapphire is also very strong, with an ultimate tensile strength at room temperature of *ca* 275 MPa. Therefore, we were particularly interested in exploring the upper limit of gas pressure that can be safely applied in such a cell, a matter not quantitatively addressed in previous work on this kind of cell.

The linear attenuation coefficient of sapphire is quite small, *e.g. ca* 0.83 and 4.4 mm^−1^ at 20 keV (λ = 0.6 Å) and 12.4 keV (λ = 1.0 Å), respectively, and therefore causes no significant absorption for capillaries of wall thickness 0.15 mm used to date, even at 12.4 keV (λ = 1.0 Å). Significantly thicker capillaries for higher working pressures can easily be accommodated by increasing the X-ray energy.

Successfully holding and sealing the sapphire capillary is crucial to the success of the experiment. This design uses Swagelok fittings for 1/16 inch tubing (1 inch = 2.54 cm), sealed with ferrules made of Vespel, which may be reused, in contrast with single-use graphite ferrules. Because sapphire is brittle, care must be taken to cut the sapphire capillary without generating cracks or micro-cracks in the pressurized region. The outer diameter of the Saint-Gobain sapphire capillaries varies by about ±(0.07–0.13) mm, so the ferrules must be carefully drilled to match the individual capillary in order to make a reliable hydrogen-tight seal. While the sapphire capillary can withstand a high internal pressure, it may easily break when twisted, owing to the brittleness of sapphire, so mechanical rigidity of the mounting arrangement and care in tightening the seals are required to avoid fracture under pressure.

The sample cell is heated by a resistive heating element composed of a tungsten filament wrapped around a 30 mm-long by 1 mm-thick quartz glass rod placed 0.5 mm under the sample (Chupas *et al.*, 2008 ▶), or by a stream of hot air. The heating element is dismounted from the sample cell when it is loaded in the glove box and remounted outside the glove box. This procedure makes the assembly of the sample cell inside the glove box much easier. The temperature is measured by a thermocouple, placed inside the sapphire capillary *ca* 1 mm from the sample and connected to a programmable temperature controller. The sample cell can be operated in the temperature range 300 < *T* < *ca* 973 K with the described setup. Alternatively, temperatures as high as ∼1273 K can be reached if the filament is wound directly around the sapphire capillary or by using two filaments (Chupas *et al.*, 2008 ▶).

### 2.3. Pressure considerations and testing the sapphire-based cell

An important question for working at elevated pressures, particularly with hydrogen, is the safe working pressure of the sapphire capillary. A safe pressure limit of *ca* 700 bar may readily be achieved for the other components, so a thin-walled capillary is the limiting factor on the working pressure of the cell. The burst pressure may be estimated from 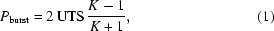
where UTS is the ultimate tensile strength of sapphire and *K* is the ratio of the outer to the inner diameter of the capillary. Equation (1) is readily derived from Barlow’s formula for circumferential stress in a thin-walled cylinder (Avallone *et al.*, 2006 ▶). Using UTS = 275 MPa (the minimum value found in the literature) and *K* = 1.38 for the 1.09 × 0.79 mm capillary referred to above, a burst pressure of 88 MPa (880 bar) is predicted, suggesting that a working pressure approaching 300 bar should be acceptable in a research situation, with a safety factor approaching 3. We point out that the safety factor is a matter of local regulation and subject to local policy on brittle materials subjected to gas pressure.

A destructive test was performed on this capillary in a purpose-built test apparatus capable of applying 2000 bar water pressure, to be described in a future publication. The design of this apparatus pays careful attention to holding the sapphire capillary without creating any residual torque or bending moment that would compromise its pressure rating through extraneous mechanical stresses. Under these conditions, the burst pressure of the capillary was found to be 900 bar, implying an ultimate tensile strength of 281 MPa, consistent with published values.

A capillary from another manufacturer that had lower visual quality (tapered, not circular) burst at a much lower pressure than predicted by equation (1). It is therefore important to use sapphire capillaries of the best possible quality, uniform in diameter and wall thickness and with low eccentricity, with the lowest possible concentration of crystal (twins, bubbles) and surface (micro-cracks) defects. It is very desirable to test to destruction a capillary from the same batch as is used for the *in situ* experiment.

### 2.4. Sample handling and preparation – the sapphire-based cell

Typically, a *ca* 10 mm length of sample is used in the sapphire cell, centred at the position of the incident X-ray beam and compacted using a 0.5 mm copper wire. The great advantage of the sample cell described here is that the material to be investigated can be loaded in a glove box and kept under an inert atmosphere. The filament heater can conveniently be mounted outside the glove box. The sample cell is equipped with an isolation valve, which is connected to the gas-handling system when the cell is mounted on the diffractometer. The double-ended setup involving the sapphire capillary can both accommodate a thermocouple and help to avoid sample displacement during rapid pressure changes. Since the gas pressure can be simultaneously applied to both ends of the sample, it allows a fast change of pressure by three to four orders of magnitude within a few seconds. The gas-handling system at MAX-lab is built from 1/16 inch standard stainless steel capillaries and fittings, regarding safety at elevated gas pressure.

### 2.5. Design of a thin-walled glass capillary-based cell

**Figure 2:**
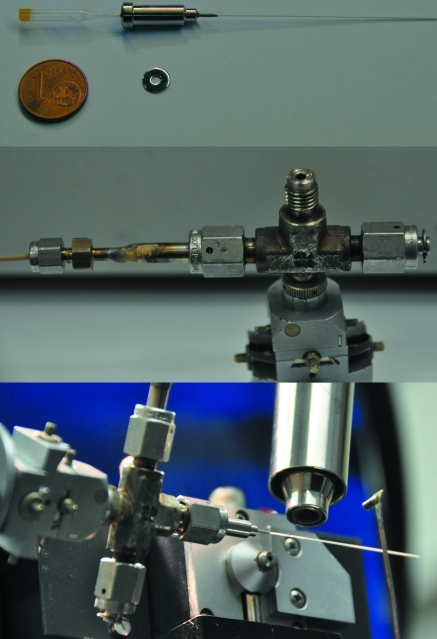
The cell based on a thin-walled glass/quartz capillary, (B). Top: the sample is loaded under Ar into a capillary closed at one end, the funnel is sealed with vacuum grease, and the capillary is glued into a socket weld gland with the funnel on the side of the VCR fitting. A 1/8 inch metal gasket is placed next to a 1 cent coin. Middle: the Tee union is fixed in the goniometer head and connected on one side *via* a PEEK capillary to a gas system; the spare outlet is closed by a cap. Bottom: the cell in operation, the nozzle of the Oxford Cryostream pointing from the top to the same position on the sample as the X-ray beam; the beamstop is shown on the right. During temperature-programmed scans, the cell is typically rotated by Δϕ = ±30° during each X-ray exposure within the same angular range.

The sample cell, shown in Fig. 2 ▶, is a modification of previously described sample cells used mainly for studies of solid–liquid hydrothermal reactions and condensation of volatile compounds, as described by Brunelli & Fitch (2003 ▶) and Norby (2006*a* ▶,*b* ▶). The cell used here is assembled from Swagelok parts. The central element is a 1/8 inch VCR Tee union (part No. S-2-VCR-T), modified in order to enable it to be fixed in a goniometric head. It is connected at one end to the gas system (Llewellyn *et al.*, 2009 ▶) *via* a very flexible PEEK (polyether ether ketone) 1/16 inch capillary from Macherey–Nagel, allowing the sample to be oscillated during data acquisition, *e.g.* Δϕ ≃ ±30°. One of the outlets of the Tee union is closed by a 1/8 inch VCR cap (S-2-VCR-CP) and the other is used for mounting the sample, as shown in Fig. 2 ▶. For that, a socket weld with a 1/8 inch VCR face seal fitting and a 1/16 inch male weld gland (S-1-VCR-3) is connected to the Tee union *via* a 1/8 inch unplated gasket (S-2-VCR-2-VS) and fixed by a 1/8 inch VCR female nut (S-2-VCR-1) from Swagelok. A capillary containing the sample is glued into a socket weld gland, with the funnel on the side of the VCR fitting. A two-component Loctite glue, Mark 3450 A and B, is used for fixing the capillary inside the socket weld gland. For good and fast solidification and low viscosity of the solidified glue under high gas pressure, the two components should be mixed close to the ideal 1:1 ratio. The use of a glued socket provides a tight and safe mounting of the capillary and allows moderately high pressures to be applied to the sample.

Glass and quartz (SiO_2_) capillaries are supplied by Hilgenberg GmbH (http://www.hilgenberg-gmbh.com/) with one end sealed. Typically, capillaries of 0.5 mm diameter and 10 µm wall thickness are used, and those made of glass have been used without fracture at room and low temperatures up to 105 bar pressure. Quartz capillaries can apparently withstand even higher pressures, subject to the absence of microcracks and other structural flaws. In order to avoid instant pressure changes that may break the capillaries, the gas system at SNBL is equipped with slow-acting valves (Llewellyn *et al.*, 2009 ▶), allowing pressure variation speeds of up to 100 bar min^−1^ in practice.

The temperature is controlled by an external calibrated cooler or heater covering the temperature range 80–500 K using an Oxford Cryostream 700+, and the high-temperature region (up to 1546 K) is covered by a Cyberstar gas blower. The distance from the nozzle of the Cryostream or gas blower to the capillary is about 4 mm, allowing a large opening angle for the diffracted beam.

### 2.6. Sample handling and preparation – the glass capillary-based cell

The present sample-cell design (B) using a Tee union has the advantage of being usable as a flow cell, when the spare opening is used as a gas outlet. In this configuration, the gas flow passes the sample and a steady state is reached. Saturation of a (few millimetres of) sample with solvent vapours transferred by a carrier gas flow typically occurs within a few seconds. The advantage is that samples with a wide distribution of particle sizes can conveniently be investigated in flow/dosing mode. In order to allow a flow of gas to pass through the sample, the sample usually needs to be sieved to obtain a narrower size distribution, preventing powder compaction in the sample.

The capillary sample holder can be loaded with powder in a glove box and sealed using vacuum grease. The funnel can then be broken off in air, sealed by a metal gasket on the Tee piece and evacuated. This procedure takes ∼15 s, provides a fast technique for handling moderately air-sensitive samples and significantly simplifies sample handling in the glove box. The effects of brief air exposure are minimized by having a few centimetres of powder in the capillary and exposing only the powder in the sealed end of the capillary to the X-ray beam. The use of the Cryostream with the thin-walled capillary cell allows the cooling of samples down to nearly liquid nitrogen temperatures. Furthermore, the price of a socket weld gland plus a thin-walled capillary is less than one-fifth of that of a sapphire capillary.

### 2.7. Gas handling – general procedure

The entire gas-handling system should be repeatedly evacuated, flushed and pressurized with an inert gas, *e.g.* nitrogen, helium or argon, to reduce the oxygen and moisture levels to a minimum and also to perform a leak test of the system, and not until then is the valve to the sample opened. The sample can be exposed to a selected gas prior to or during the X-ray experiment. The sample holder, whether made of glass or single-crystal sapphire, is clearly the weak point in the cell-plus-gas handling system. Minor fractures or micro-cracks in the sapphire single-crystal capillary or the glass capillary greatly reduce the burst pressure. Therefore, the sample container should be inspected before use and those with defects should be discarded. The gas-handling system can be made mainly of standard stainless steel components and should be constructed so that the gas volume is minimized. Furthermore, the gas cylinder should be kept closed at all times during X-ray experiments, except when the gas pressure in the volume attached to the sample is adjusted. The isolation of the gas supply cylinder reduces the gas release to the small quantity present in the pipes (*e.g.* diameter 1/16 inch) and sample holder in case of leaks.

## 3. Experimental results

A standard experiment aimed at studying properties and transformations in hydrogen storage materials involves hydrogen desorption in vacuum or under a certain hydrogen pressure, *e.g. p*(H_2_) = 1–5 bar, and possibly rehydrogenation under various gas pressure and temperature conditions. These experiments are typically performed using *in situ* diffraction on polycrystalline powders.

In order to test the sapphire sample-cell design (A) and to demonstrate the study of fast solid–gas reactions, a sample consisting of magnesium hydride, MgH_2_, ball-milled with scandium chloride, ScCl_3_, was prepared and studied by *in situ* synchrotron radiation powder X-ray diffraction (SR-PXD).

Powdered MgH_2_ and ScCl_3_ were purchased from Aldrich and used as received. A mixture of MgH_2_ and ScCl_3_ (5.0 mol%, 23.2 wt%) was placed in a tungsten carbide vial under an inert atmosphere. The vial was sealed and transferred to a Frisch Pulverisette 4 and milled for 30 min with a ball-to-powder mass ratio of 41:1. All sample handling was performed under a purified argon atmosphere in a glove box.

**Figure 3:**
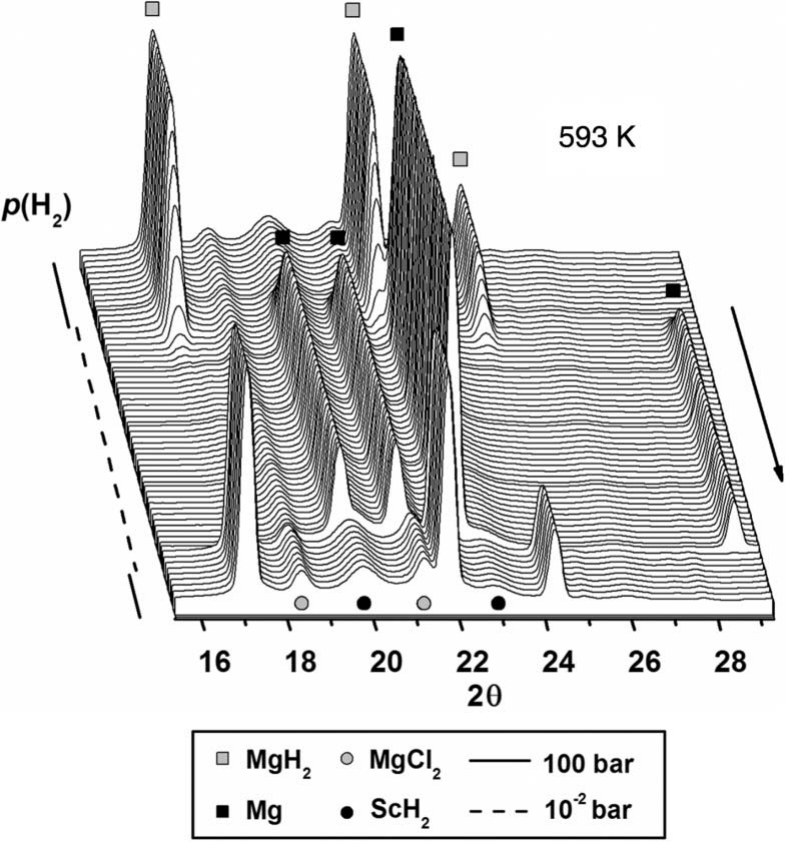
Isothermal hydrogen release and uptake reactions studied for a sample of magnesium hydride ball-milled with scandium chloride (5.0 mol%). The SR-PXD data were collected using the sapphire-based cell (A) at a fixed temperature of 593 K for a period of 30 min and the hydrogen pressure varied in the range ∼10^−3^ to 100 bar. The X-ray exposure time was 5 s, the time resolution was 9.95 s per powder pattern and the selected X-ray wavelength was λ = 0.94608 Å.

The sample was studied by *in situ* SR-PXD experiments at beamline I711 at MAX-lab, Lund, Sweden. The sample was loaded into a sapphire capillary and mounted in the cell under inert conditions. Data were collected in the temperature range from room temperature to 723 K (heating rate 15 K min^−1^), and also with the temperature kept fixed at 593 and 623 K for 30 and 20 min, respectively. The hydrogen pressure varied in the range ∼10^−3^ to 100 bar. The X-ray exposure time was 5 s, giving a total of *ca* 1200 PXD profiles measured using a selected wavelength of λ = 0.94608 Å. The time resolution of the X-ray diffraction experiment is the sum of the exposure time and detector read-out time, which amounts to 9.95 s. The diffraction profiles are summarized in Fig. 3 ▶.

The *in situ* SR-PXD data shown in Fig. 3 ▶ reveal that the compounds MgH_2_ and ScCl_3_ reacted during ball-milling to form magnesium chloride MgCl_2_ and scandium(II) hydride. This shows that Sc^III^ is reduced to Sc^II^, and that ScH_2_ appears to be the catalytically active compound mediating fast hydrogen uptake by and release from Mg/MgH_2_. There is no observable change in the diffracted intensity from ScH_2_, suggesting that it is chemically unchanged during hydrogen release and uptake, and indicating that it may have a purely catalytic role. In this study, a relatively large amount of catalytically active additive (5 mol% ScCl_3_) was used in order to make a possible chemical reaction more clearly observed by PXD. It has been found that smaller amounts of transition metal additives also act as a catalyst for H_2_ release and uptake in the MgH_2_/Mg system, *e.g.* 0.5 mol% Nb_2_O_5_ (Barkhordarian *et al.*, 2004 ▶).

**Figure 4:**
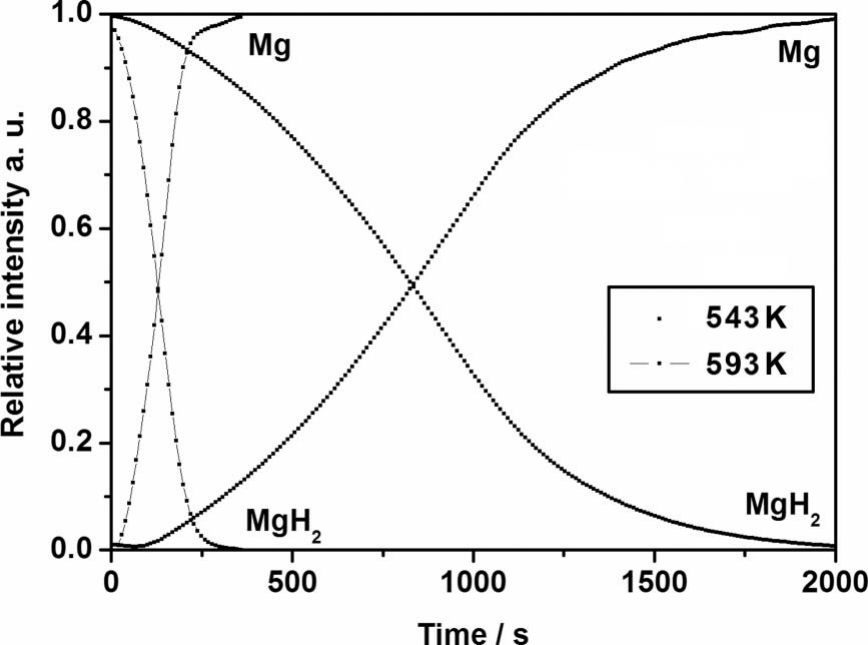
Integrated normalized diffracted intensity, α(*t*), from Mg and MgH_2_ at 543 and 593 K during release and uptake of hydrogen. The PXD data were collected using the sapphire-based cell (A) and extracted from Fig. 3 ▶.

The diffracted intensity from Mg and MgH_2_ can be integrated and converted to approximate phase fractions by normalizing the intensities, α(*t*) = *I*(*t*)/*I*_o_, where *I*_o_ is the maximum diffracted intensity (Fig. 4 ▶).

The phase fraction curves α(*t*) for Mg and MgH_2_ intersect at α ≃ 0.5, which shows that the reaction does not involve a (rate-limiting) reaction forming an intermediate phase. *In situ* SR-PXD data can be useful for kinetic investigations and can provide apparent activation energy and rate constants from isothermal gas release or uptake data. Kinetic models such as Avrami or ‘exponential growth’ models are often applied (*e.g.* Myhra *et al.*, 1995 ▶; Christensen *et al.*, 1998 ▶; Jensen *et al.*, 2006 ▶). Indications of the rate-limiting processes can also be obtained in some cases (Bösenberg *et al.*, 2010 ▶).

**Figure 5:**
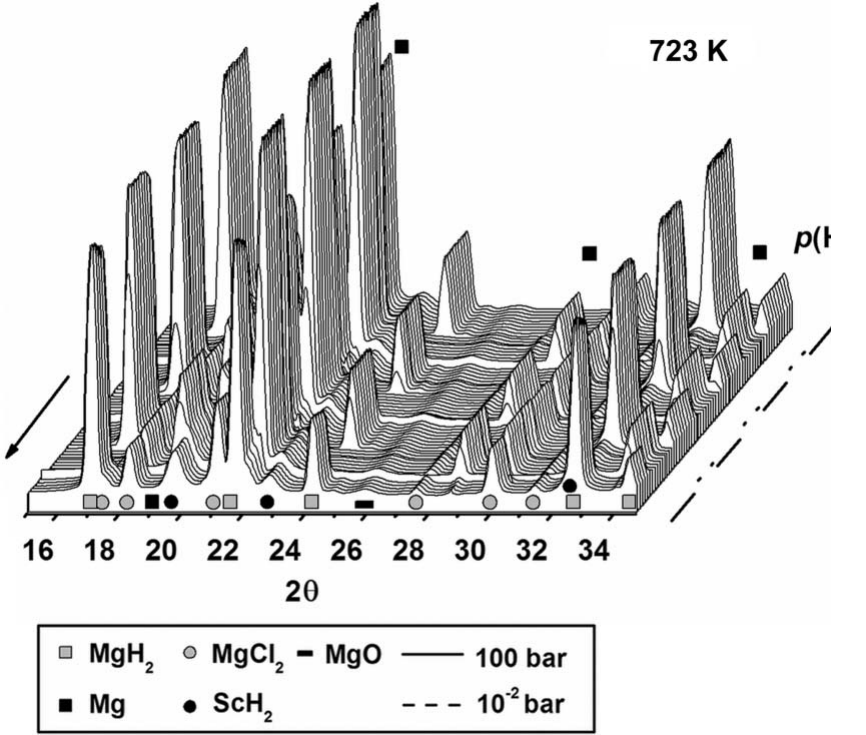
Isothermal hydrogen release and uptake reactions studied for a sample of magnesium hydride ball-milled with scandium chloride (5.0 mol%). SR-PXD data were collected at 723 K over a period of 20 min, performed as a continuation of the experiment shown in Fig. 3 ▶. The PXD data were collected using the sapphire-based cell (A) using an X-ray exposure time of 5 s, a time resolution of 9.95 s per powder pattern and λ = 0.94608 Å. The hydrogen pressure was either ∼10^−2^ or 100 bar.

The SR-PXD data shown in Fig. 5 ▶ were measured using the sapphire cell (A) under isothermal conditions, varying the hydrogen pressure between ∼10^−2^ and 100 bar. The change in hydrogen gas pressure resulted in drastic changes in the sample composition, which was partly resolved using the selected exposure time and a time resolution of ∼10 s per powder pattern. The gas pressure was changed every 3 min.

**Figure 6:**
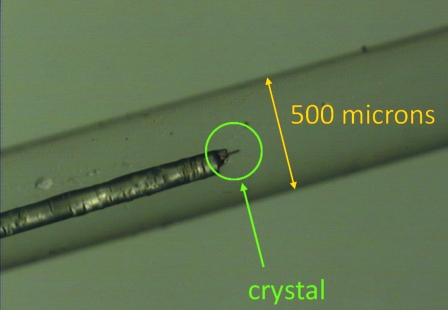
A single crystal mounted on a needle and fixed inside a thin-walled capillary tube using sample holder (B). This setup allows single-crystal diffraction studies under a gas loading (Miller *et al.*, 2009 ▶).

The versatility of the two capillary sample holders is further illustrated by their use for studies of gas adsorption in single-crystal samples. A single-crystal metal–organic framework compound was mounted on a thin glass needle, which in turn was placed inside the capillary and fixed to its wall by a droplet of glue using sample holder (B) (see Fig. 6 ▶). Such diffraction data allow the location of gas molecules like CH_4_ and CO_2_ inside the pores of the solid material (Miller *et al.*, 2009 ▶). It is more challenging, however, to obtain the position and quantity of lighter physisorbed gas molecules such as nitrogen and hydrogen even from single-crystal diffraction data. Such experiments require cryogenic conditions.

**Figure 7:**
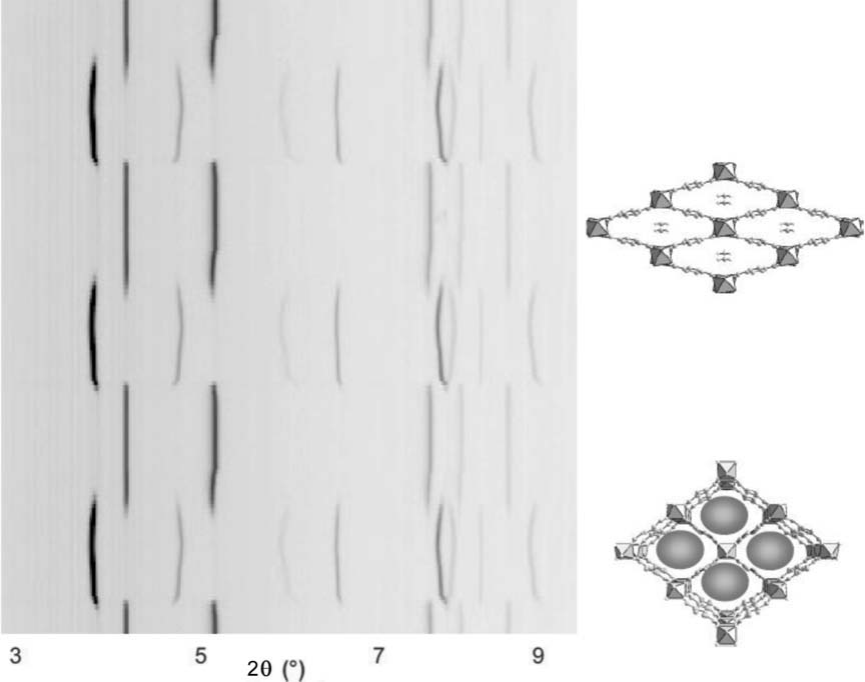
The cyclic behaviour of CO_2_ absorption by a flexible metal–organic framework (Serre *et al.*, 2007 ▶). Closed and open forms, shown schematically on the right, are reversibly transformed into each other as the gas pressure periodically varies from 0 to 10 bar (vertical scale). SR-PXD data collected *in situ* using the capillary cell (B) are shown on the left.

The described gas cells (B) were also used to study the cyclic behaviour of gas absorption in metal–organic frameworks (Serre *et al.*, 2007 ▶) and transformations in coordination polymers triggered by dehydration/rehydration (Sereda *et al.*, 2009 ▶). In the first case, the pressure of pure CO_2_ gas was periodically varied between 0 and 10 bar, observing an associated opening/closing of the flexible framework (Fig. 7 ▶). In the second type of experiment, flows of a wet (containing water vapour) and dry neutral gas were alternated, leading to a reversible transformation associated with hydration/dehydration of a coordination polymer.

## 4. Discussion

The two sample cells discussed in this paper are optimal under somewhat different conditions, and thus may be considered complementary. The advantage of the sapphire-based cell (A) is first of all related to its higher pressure limit of 300 bar. This cell also shows no amorphous contribution to the background. Besides a higher background, glass or quartz capillaries show a broad hump at ∼3.6 Å. Although the high statistics of two-dimensional detectors allow suppression of the noise and the satisfactory modelling of the background, the amorphous contribution of the sample holder can be a major part of the scattered intensities such as for light hydrides, *e.g.* LiBH_4_ (Filinchuk, Chernyshov & Černý, 2008 ▶). Another advantage of sapphire is its chemical inertness, *e.g.* towards molten borohydrides (Mosegaard *et al.*, 2008 ▶). The sapphire cell can also be used for studies of crystallization under supercritical conditions (Bremholm *et al.*, 2008 ▶). In contrast, glass and quartz can react with solid hydrides such as magnesium hydride at elevated temperatures (Jensen *et al.*, 2006 ▶).

An advantage of the system based on capillaries, (B), fed *via* a flexible PEEK capillary is the larger angle of possible capillary rotation (typically ±30°), which improves the powder averaging even if the sample contains larger crystallites, typically producing ‘spotty’ diffraction rings: recrystallization and particle growth often occur during phase transitions or reactions in the sample, *e.g.* during de- and rehydrogenation of hydrides. On the other hand, diffraction spots from the single-crystal sapphire capillary itself may be avoided by rotating the sample a few degrees prior to the X-ray experiment; sample rotation during a diffraction experiment increases the probability of also measuring diffraction from the sapphire.

Both sample cells are versatile and can also be used for studies of solid–liquid–gas reactions. Hydrothermal decomposition experiments on gypsum were performed by grinding the solid material, CaSO_4_·2H_2_O, with water or aqueous solutions of either 1.0 *M* HNO_3_ or 1.0 *M* LiCl in a mortar. The suspensions were injected into sapphire capillaries using a syringe and heated to 463 K in a nitrogen gas pressure of *p*(N_2_) ≃ 13 bar in order to suppress the boiling of the suspensions and to allow *in situ* SR-PXD data to be recorded for the hydrothermal decomposition of gypsum in different liquids (Christensen *et al.*, 2008 ▶).

The quartz glass sample cell (B) also allows for *in situ* mixing of a solid with a liquid during data acquisition, which was done for a study of the hydration of α-CaSO_4_·0.5H_2_O in water. A quartz glass capillary (0.7 mm outer diameter) was loaded with solid α-CaSO_4_·0.5H_2_O compacted to ∼2 mm and placed in a vertical position. Water was introduced into the capillary with a syringe, so that the meniscus was *ca* 2 mm from the dry sample. A few diffraction patterns of the dry sample were measured at 298 K, prior to increasing the pressure in the capillary to *p*(N_2_) ≃ 10 bar. This brings water into contact with the dry sample *in situ* during data acquisition, and this technique allows studies of fast solid–liquid reactions (Christensen *et al.*, 2004 ▶, 2008 ▶; Jensen *et al.*, 2005 ▶).

The very small amount of sample present in the sapphire capillary cell (A) makes measurement of the absorbed/desorbed hydrogen quantity by the manometric Sieverts technique (sharing the gas between a calibrated volume and the sample cell) difficult but not impossible, given a sufficiently small total system volume, excellent temperature control and an excellent pressure transducer (Gray, 2008 ▶). This option will be explored further in the near future, when we hope to achieve the same facility with milligram samples in a capillary presently available with larger samples (Gray *et al.*, 2006 ▶).

## 5. Conclusions

In this paper multipurpose sample cells for *in situ* PXD are presented, along with selected studies within hydrogen storage research. A major advantage is the excellent time resolution and fast change of pressure that allow the study of hydrogen release and uptake in real time for fast reactions. Some important practical considerations are discussed, particularly the maximum achievable working gas pressure that can be achieved with a thin-walled sapphire sample capillary. It is found by destructive testing that the 1.09 × 0.79 mm single-crystal sapphire capillary commonly used in our experiments has a burst pressure of *ca* 900 bar, commensurate with a working pressure of several hundred bar. This cell also has the potential for simultaneous Sieverts measurements of gas uptake and release, and *in situ* SR-PXD studies of structural changes and changes in phase composition. An alternative sample cell based on a thin-walled glass or quartz capillary, connected to a hydrogen source *via* a VCR fitting, enables studies up to 100 bar. Both cells are versatile and capable of solid–gas and solid–gas–liquid investigations.
